# Finding Meaning: HIV Self-Management and Wellbeing among People Taking Antiretroviral Therapy in Uganda

**DOI:** 10.1371/journal.pone.0147896

**Published:** 2016-01-25

**Authors:** Steve Russell, Faith Martin, Flavia Zalwango, Stella Namukwaya, Ruth Nalugya, Richard Muhumuza, Joseph Katongole, Janet Seeley

**Affiliations:** 1 School of International Development, University of East Anglia, Norwich Research Park, Norwich, United Kingdom; 2 Medical Research Council / Uganda Virus Research Institute, Uganda Research Unit on AIDS, Entebbe, Uganda; 3 External Research Associate, School of International Development, University of East Anglia, Norwich, United Kingdom; 4 London School of Hygiene and Tropical Medicine, London, United Kingdom; Liverpool School of Tropical Medicine, UNITED KINGDOM

## Abstract

The health of people living with HIV (PLWH) and the sustained success of antiretroviral therapy (ART) programmes depends on PLWH’s motivation and ability to self-manage the condition over the long term, including adherence to drugs on a daily basis. PLWH’s self-management of HIV and their wellbeing are likely to be interrelated. Successful self-management sustains wellbeing, and wellbeing is likely to motivate continued self-management. Detailed research is lacking on PLWH’s self-management processes on ART in resource-limited settings. This paper presents findings from a study of PLWH’s self-management and wellbeing in Wakiso District, Uganda. Thirty-eight PLWH (20 women, 18 men) were purposefully selected at ART facilities run by the government and by The AIDS Support Organisation in and around Entebbe. Two in-depth interviews were completed with each participant over three or four visits. Many were struggling economically, however the recovery of health and hope on ART had enhanced wellbeing and motivated self-management. The majority were managing their condition well across three broad domains of self-management. First, they had mobilised resources, notably through good relationships with health workers. Advice and counselling had helped them to reconceptualise their condition and situation more positively and see hope for the future, motivating their work to self-manage. Many had also developed a new network of support through contacts they had developed at the ART clinic. Second, they had acquired knowledge and skills to manage their health, a useful framework to manage their condition and to live their life. Third, participants were psychologically adjusting to their condition and their new ‘self’: they saw HIV as a normal disease, were coping with stigma and had regained self-esteem, and were finding meaning in life. Our study demonstrates the centrality of social relationships and other non-medical aspects of wellbeing for self-management which ART programmes might explore further and encourage.

## Introduction

In Uganda people living with HIV (PLWH) on antiretroviral therapy (ART) with access to good treatment support can have the same life expectancy as the general population [1]. However HIV remains a challenging condition to manage, for example due to poverty, stigmatisation, regular health care visits and the difficulties posed for people’s sexual and reproductive lives [2,3].

The health of PLWH and the sustained success of ART programmes depend not only on the health care available but also on PLWH’s motivation and ability to self-manage the condition, including daily adherence to drugs. Although there is a growing body of evidence about ART adherence and its influences in resource-limited settings [4–7], there is still relatively limited research on the range of PLWH’s self-management processes on ART in resource-limited settings [2,8,9]. This paper aims to enhance knowledge and understanding of HIV self-management processes in one particular resource-limited setting, Uganda. Analysis extends beyond adherence to examine a range of social, psychological and practical self-management processes, which in turn are closely related to and influence PLWH’s wellbeing.

Self-management involves a range of processes [10–12] which derive from a person’s work to sustain wellbeing [13], to incorporate the illness and treatment into one’s identity and practical life [9], and more specifically in psychological terms “…to maintain a positive view of the self and the world in the face of a health problem” (11: 1161). Systematic reviews of self-management inform our analytical framework [10,11] and we refer to three broad domains of self-management, and within each more specific processes and tasks:

- Mobilising resources (for example from health care providers and social networks)
- Managing illness needs (for example adhering to drugs, eating nutritious food)
- Living with the condition (for example adjusting to the illness, adjusting to the new ‘self’, finding meaning).

Dimensions of wellbeing are also analysed because they overlap with or derive from self-management. Successful self-management of HIV can sustain physical and psychological wellbeing, and wellbeing is likely to motivate self-management [14]. A few studies in sub-Saharan Africa are surprisingly reporting *higher* quality of life among PLWH on ART compared to their HIV-negative counterparts [15–17]. Quantitative findings from our own study in Uganda add to this evidence, showing higher self-reported quality of life and lower depression scores among PLWH compared to a community control sample [18]. These counter-intuitive findings are potentially explained by PLWH’s better access to quality health care compared to the HIV negative sample [16], and possibly greater social contact created by regular visits to the clinic [18,19]. An additional aim of this paper is therefore to examine possible factors explaining relatively high quality of life scores among PLWH on ART, using people’s narratives about their own self-management and subjective wellbeing.

We use White’s [20] conceptualisation of wellbeing, which has similarities to the quality of life concept. It is based on the material, relational and subjective domains of life which make up what people have, what they can do and be, and what they think or feel. The material domain includes conventional and ‘measureable’ dimensions of wealth or deprivation, such as income, asset or nutrition levels. The relational domain is divided into two: close or personal relationships with others; and wider social divisions and inequalities in society (for example based on gender), and the access to goods or services, forms of domination, and sense of connectedness to the community arising from these social relations [20]. The material and relational domains of wellbeing have subjective dimensions as well as ‘objective’ (observable, verifiable) ones, for example how satisfied people are with their income, their relationships or their experience with services, their self-esteem, and whether they feel a sense of belonging in the community.

Subjective wellbeing is not necessarily lowered following serious illness or disability because people make psychological adjustments to their expectations. In this paper we refer to the linked adjustment processes of ‘response shift’ and ‘contrast effects’. Response shift theory refers to three-related changes in how a person assesses what constitutes a good life when faced with serious illness [12,21]. First, after being very sick with HIV, a person might give a higher value to ‘normal health’ on ART than they would have done before being ill (recalibration). Contrast effects contribute to this recalibration of standards. For example a person who judged a state of physical functioning as ‘poor’ *before* they fell seriously ill may afterwards judge this state as ‘good’ [22]. Second, the relative importance of health (with ART) compared to other life domains might increase (reprioritisation). Third, wellbeing might be more radically reconceptualised as people add or remove life domains from their subjective assessment of wellbeing, for example finding a new purpose to life or meaning in religion.

Self-management interventions for HIV in sub-Saharan Africa have not been well researched, and it is not clear how such interventions need to be adapted [23]. Our analysis seeks to highlight the factors which contribute to effective self-management of HIV and an enhanced sense of wellbeing. Better understanding of what helps people self-manage and what enhances wellbeing can help us know how to adapt ART programmes to meet people’s needs.

## Materials and Methods

### Research design and study site

In 2011–12 qualitative and quantitative data were collected for a study on people’s self-management of HIV and wellbeing in Wakiso District, Central Uganda. Participants were recruited from three types of ART delivery site in the district: the HIV clinic at a government hospital; three government health centres that have referral links to that hospital; and a branch of a well-established non-governmental organisation, The AIDS Support Organisation (TASO). In this paper we present the qualitative findings about 38 PLWH’s self-management and wellbeing on ART. The quantitative measures of quality of life and depression outcomes among a larger sample of PLWH and a community control sample are presented elsewhere [18].

Ethical approval for the study was obtained from the following ethical review boards: the Uganda Virus Research Institute Science and Ethics Committee, in Uganda, and; the International Development Research Ethics Committee, University of East Anglia, in the United Kingdom. Overall permission to conduct the research was obtained from the Uganda National Council for Science and Technology. Written informed consent was obtained from all participants in the research. Pseudonyms are used in this paper to maintain confidentiality.

### Sample

To be eligible participants must have been on ART for more than one year. A list of eligible patients was compiled for each facility. The lists contained hundreds of patients, so to reduce the number from which we would purposefully sample a systematic random sample was first taken using intervals to generate twice the number of cases required, anticipating refusals or early drop outs. These lists were then stratified by age and gender, and 42 participants were purposively sampled from the gender and age categories to ensure gender balance, a mix of ages, and a range of patient experiences.

### Data collection measures

Two in-depth interviews were conducted with each participant. The first was an unstructured life and illness history, and due to the wide-ranging and sensitive nature of the questions, these data were collected over one to three visits, depending on how long the participant could spend with the researcher on each visit. The first interview(s) were not recorded, because experience in this setting indicated people are more open when not being recorded, especially in the first few interviews, but notes were taken and detailed life and illness history narratives were written up in English by the interviewers.

The second interview was semi-structured, and this was recorded, transcribed and translated into English. The question guide was informed by issues raised in the first life history interviews, as well as the research objectives. These interviews explored participants’ approaches to self-management and changes in their lives and wellbeing since becoming HIV positive and starting ART. The interviews explored people’s experiences and how they felt about their activities and achievements (or disappointments). The use of more than one visit to meet participants allowed a degree of trust to develop, which in many cases led to rich discussions of participants’ experiences.

### Analysis

Qualitative data were organised using QSR Software NVIVO 9. To ensure analytical rigour, two researchers independently coded and checked results. Thematic interpretations of the data were discussed in more detail by the team at a two-week analytical workshop in Uganda. Themes were tested further by checking counter examples and exceptions. Quotes used in the paper are either the words of the participants or the interviewer’s words used in the write up of the first interview. Frequently used expressions used by participants across the interviews are not quoted but set out using italics.

## Results

Thirty-eight participants were included in the final analysis because of four incomplete interviews. We briefly summarise the socio-economic circumstances of the 38 participants because meeting minimum needs is a foundation of wellbeing [24]: if minimum consumption needs cannot be achieved for example, this is likely to undermine self-management and subjective wellbeing. The three domains of Schulman-Green et al’s [10] self-management framework are then analysed.

### Self-management and poverty

Table 1 summarises the socio-economic characteristics of the 38 participants (20 female; 18 male). The majority of participants (22/38) were aged between 26–40 years, 14 participants were aged 41–60, and two were over 60 years old. Thirteen were recruited from the government hospital, 11 from the three referral health centres, and 14 from TASO.

**Table 1:** Participant socio-economic characteristics.

Gender / relationship	Material wealth status (categories derived from observation and narratives)	Name (age)
**Single women**	Financially independent, adequate income	Happy (46)
		Grace (32)
		Linda (29)
		Hannah (46)
	Dependent on family	Gloria (29)
		Ruth (58)
	Getting by	Naome (26)
		Joy (27)
	Income poor	Ann (29)
		Nana (46)
		Suzan (43)
	Extremely poor	Mercy (35)
		Jackie (61)
**Women with a partner**	Adequate income	Dorcas (42)
		Paloma (31)
	Income poor	Sarah (38)
		Ritah (39)
		Julie (37)
		Judith (27)
	Extremely poor	Bridget (33)
**Single Men**	Adequate income	Aaron (40)
		Tom (44)
	Income poor	Davis (43)
		Vincent (74)
	Extremely poor	Simon (30)
**Men with a partner**	Adequate income (although money worries still expressed)	Benson (34)
		Jerry (45)
		Dominian (38)
		Peter (38)
		Matthew (51)
		Mark (31)
		Jacob (32)
		Derrick (38)
	Income poor	Paul (39)
		Fred (47)
		Bridge (42)
		Isaac (38)
	Extremely poor	Angelo (47)

More than half the participants had some primary education and the majority were married or in a relationship. They engaged mainly in small scale farming, fishing, building and petty trade. Nearly half were income poor (8/18 men; 10/20 women), and with differing frequency struggled to meet basic food needs. The others had an adequate material standard of living through their salaries or successful businesses such as fishing, housing for rent and poultry farming. Those able to cultivate around their homes were usually able to eat one meal a day, but a small minority in extreme income poverty faced a daily struggle for enough food. Several participants said that inadequate food intake made taking the drugs more difficult.

For the majority it was poverty, rather than HIV, that was the main stressor that sometimes undermined feelings of wellbeing, and was a cause of *too many thoughts* (an expression used to describe stress or low mood):

“What worries me so much is the (financial) situation we are in as you see. I have a big family…but…I sometimes worry and ask is this child going to be chased away from school, what are we going to eat, and this gives you sleepless nights… But if it was not for that, my heart would have been settled and I have everything, I would be okay…” (Peter, male (M), 38).

Despite economic hardships the recovery of health and hope with ART appeared to have strengthened the participants’ ability to cope psychologically with the difficulties and worries of poverty and their condition. The majority had a determination to take the drugs, even when food was lacking, because they valued their new chance at life:

“Even if he does not have the food, he would rather take the drugs in pain because they are more valuable to his life than the food…” (Paul, M, 39).

Participants were managing their condition well across the three domains of self-management, and the majority (37/38) had become proud adherers to ART, indicated by their health and their sense of wellbeing. This contrasts with their situation before ART, when 31 of the 38 participants had been suffering from serious opportunistic infections, and some had been close to death. At the time of the study only one man was not adhering well to treatment, possibly because of mental illness.

The majority of the participants’ sense of self-efficacy and their successful self-management is unlikely to be typical of all PLWH in this setting. They had tested, were adhering to ART, and although the majority were cautious about disclosure, they were open enough about their status to be willing to participate in the research. Not all PLWH in settings like this can self-manage effectively on ART or achieve the levels of wellbeing evidenced here (McGrath et al. 2014).

### Mobilising resources

#### Good relationships with health workers

In Uganda, HIV service infrastructure has received substantial funding since 2004. Treatment and counselling are available from government and non-government providers which is often not the case for other conditions. Participants praised the fact that they could get life-saving ART free at the point of delivery, the drug consistently referred to as *the most important thing that has helped me to cope*, and *the drug that makes the difference*.

Positive relationships with health workers were a critically important resource for the participants (Table 2), especially in the first months following diagnosis and starting treatment. A powerful theme in the narratives was participants’ enthusiasm for the caring and respectful approach of health workers, and the good relationships they had built with them.

“The health workers at the hospital clinic were very warm and welcoming, which gave her courage to…keep going back” (Ruth, Female (F), 58).

“When I went to TASO, I felt like I was with my friends” (Naome, F, 26).

“When I came to the clinic they showed me love…if they had not encouraged me I would be dead…” (Judith, F, 27).

**Table 2:** Mobilising and building resources.

Self-management resource	Self-management process or skill and related wellbeing dimensions
**Using the health care system**	Access to treatment, and satisfaction with HIV services
	Collaborative and respectful relationships with health workers, providing resources of knowledge, practical skills and new ways of thinking and talking about HIV
**Spiritual and social resources**	Drawing courage and strength from God, putting worries aside and placing the unknowns in God’s hands
	Being part of a spiritual community
	Obtaining emotional and material support from close family members
	Peer support among fellow patients: a sense of shared experience, solidarity and group identity

One participant noted the stark contrast between the quality of care which they received at the ART clinics compared to the ‘usual services’ in the health system:

“The way our *Basawo* (health workers) treat us, it is not the same as those who administer treatment for malaria or fevers. They treat us like people…they counsel us and they take care of us. They (health workers who give other treatment) are really difficult people…they even slap you” (Jerry, M, 45).

The narratives revealed how good relationships with health workers had enhanced receptiveness to three inter-related resources for self-management (Russell et al., 2015):

- Knowledge about the condition
- Practical advice and skills for self-management
- New concepts and language for coping

The participants drew on this knowledge and advice to support their work at self-management across the three domains (Table 2). As a first step, participants recalled how health workers had given them knowledge which helped them reconceptualise HIV as a controllable condition with ART, rather than a ‘death sentence’. Common expressions used to describe how they first felt after diagnosis included: *my life is over*, *you know you are going to die*, *who will care for the children*. Some participants’ worry was so intense that they felt they would die of worry before HIV. Acquiring knowledge about HIV and ART from health workers was a great psychological resource and emotional ‘boost’: it reduced uncertainty and anxiety, provided hope, and gave them an inner strength to carry on. They could start to see the possibility of regaining control over their health and their lives again:

“My heart became strong because of the things that we were told by the health workers” (Judith, F, 27).

#### Spiritual and social resources

Spiritual and social resources helped psychological coping and wellbeing (Table 2). In a context where religiosity is widespread many participants felt God was looking over them and would take on the responsibility, and the stress, of any uncertainty about their health and when they might die. God as a supporter and protector helped sustain psychological wellbeing, despite adversity:

“God loves me so much and he knows that I don’t have anyone who can support me apart from Him. That is why he has protected me from getting bedridden, keeping me strong and able to take myself to the hospital…every day before going to bed, I must thank him for the gift of life” (Hannah, F, 46).

Emotional support from one or two close family members, usually the person(s) to whom the participant had first disclosed, was also important and several participants stated that one particular person was *the reason I am still here*:

“Mother at first cried and said that God you have decided to take all of my children with HIV but later on she told me to be strong. My older sister who is HIV positive was around so she comforted me and counselled me and that helped me so much” (Gloria, F 29).

Encouragement from a new network of friends from the HIV clinic was a prominent theme. The participants spoke about TASO and government facilities as a space, and their regular appointments as a dedicated time, in which they could have caring interactions not just with staff, but with other PLWH, to share experiences and encourage each other. Female participants talked more about the benefits of finding new friendship groups at service providers:

“This is a great feature about meeting people at the clinic…we also give each other a call to check up on each other and things like that…we are encouraged because we are not alone; so many others are ill” (Bridget, F, 33).

These shared experiences encouraged self-management and enhanced wellbeing because they helped create a sense of membership and relatedness, of solidarity, an ‘in-group’ belonging to a ‘therapeutic community’:

“When we are gathered at the clinic, we benefit a lot. This situation unites us and we are the same. In fact, we call ourselves members; so when we meet, we simply greet each other with ‘hello member’. It is as if we are in a club” (Tom, M, 44).

The caring relationship participants felt that they had with their doctor or counsellor, a person who listened, was also an important part of their emotional support network. The psychological benefits of counselling extended beyond HIV and ART topics, to other areas of life:

“Counselling is good, it could be about anything—husbands, medication, food, money. … just to talk to someone” (Bridget, F, 33).

Good social relationships and a sense of connection are important sources of wellbeing in their own right, and can also enhance psychological coping at times of stress which sustains subjective wellbeing, for example after the onset of serious illness [24]:

“Having people around you is enough to recover” (Ruth, F, 58).

### Managing illness and physical health

#### Following the practical advice, taking ownership of health

All the participants enthusiastically spoke about the practical advice and ‘instructions’ that health workers had given them to manage their health. It provided a very useful structure both to manage their health and to live their life:

“I knew that I was finished and was just waiting for the day I would die. But sticking to the words of the *basawo* (health workers) has helped me…and that is why I look fine now” (Jerry, M, 45).

Positive relationships with health workers encouraged participants to listen and absorb this practical advice (Table 3). It also encouraged continuing engagement with treatment:

“The health workers at the hospital clinic were very warm and welcoming, which gave her courage to remain there and keep going back” (Ruth, F, 58).

**Table 3:** Managing illness needs and physical health.

Self-management task	Self-management process or skill and related wellbeing dimensions
**Learning about HIV and performing health promotion activities**	Using the practical advice and knowledge from health workers to manage health
**Taking ownership of health needs and completing health tasks**	Becoming an expert: confidence and sense of self-efficacy developed
	Adhering to ART, despite difficulties
	Gaining a sense of control over one’s health and life
**Achievements which sustain motivation to self-manage and enhance wellbeing**	Sense of pride and achievement from adhering to drugs
	Improved physical health and functioning
	Contrast effects (before and after) enhance subjective wellbeing

Health workers’ power or authority did not appear to have undermined participants’ sense of agency, or in a Foucaultian sense fashion ‘docile patients’ through control and discipline. On the contrary they argued the framework, delivered in a respectful way, had supported their own adjustment to life on ART, it gave them back a sense of control over their lives, summed up by this female patient:

“I help myself the most with my illness, because I follow the instructions of the health workers” (Gloria, F, 29).

A male participant articulated the sense of control and security he felt from the instructions:

“I am in control of the illness when I respect what the health workers tell me to do. …That is why you see me build and going forward. …If you ask me my dreams now or what I am planning, I can tell you that I am going to plant a mango tree and I will be able to eat fruits from it (Peter, M, 38).

The health worker-patient relationship was therefore productive for this group of PLWH, even though it was hierarchical. Participants had made their own decisions to ‘follow the rules’, and felt able to make decisions about when and how to transgress the behavioural boundaries for self-management set by the health workers. They were adhering to the drugs, but over time they negotiated the rules to suit their needs or other pressures, especially around decisions about condom use, whether to try and have children, and social drinking [25]. For example two of the women had had a child since starting ART and some men continued to have sexual partners outside marriage, although *they no longer went with so many women* and used condoms outside marriage. One man chose not take the drugs on Friday evenings because he went out to socialise and drink beer with friends: this selective ‘non-adherence’ was part of his own self-management and search for wellbeing, balancing health and social life.

#### Pride in achievements

Participants derived a great sense of achievement and pride from following the instructions and seeing the results of their efforts. They felt good about their competence in managing the condition:

“In the past there was so much fear (about HIV)…(But now) I drink my beer and I tell the people around that I am HIV infected, and I am proud…I show off because I look good” (Mark, M, 31).

One other achievement emphasised by participants was how well and normal they *looked* as well as felt. Looking ‘normal’ had significant benefits for personal and social confidence and self-esteem: it meant they could pursue life ‘like a normal person’. This sense of social inclusion was vital for their wellbeing and enhanced further by the pleasure they got from seeing people’s surprise at how well they looked:

“When people see him now, they exclaim that he has a powerful engine. This is because he is strong and can now work and do anything. These are the same people who were saying that he was going to die” (Peter, M, 38).

The participants enthusiastically made stark contrasts between their health before and after ART. Their recovery appeared to have had a particularly powerful positive contrast effect for wellbeing: they no longer took their health for granted. They had recalibrated their standards of health (a dimension of ‘response shift’), giving more value than they would have done before to ‘feeling normal’. They had also given greater priority to their health over other domains of life.

### Living with HIV on ART

#### Adjustment to and ‘normalisation’ of HIV

Living with HIV on ART is a domain of self-management involving work to ‘adjust’ to the condition (Table 4).

**Table 4:** Adjusting to life with HIV on ART.

Self-management process	Self-management process or skill and related wellbeing dimensions
**Adjusting to illness emotionally, cognitively, practically**	‘Normalising’ HIV, a new language to think and speak about HIV
	Re-establishing activities and ‘normal’ social roles: working again, parenting again, re-connecting to the social life of the community
	The therapeutic value of work
**Adjusting to ‘new’ self**	Reframing the self as normal
	Managing and resisting stigma, making social comparisons: ‘we are the responsible ones’
**Finding meaning**	Greater appreciation of life and health
	A new or clearer sense of purpose, supporting and advising others, working hard for the future and for children’s future
	Stronger relationship with God

Health workers had helped processes of adjustment by giving participants a new language for thinking and speaking about HIV, helping participants reconceptualise their condition as ‘normal’, *like many other diseases*, and also as one of many causes of death: *you can die from many other diseases and many other things; death comes to us all*, *so how is HIV any different*? HIV was also reconceptualised as a normal disease by referring to its wide prevalence across the community. From the time of diagnosis, health workers had told participants *you are not alone*, *look around you*. All the participants drew on this language, using the phrases *I am not the only one*, *I share this problem with many others*:

“We are very many on drugs in this area…we are many and they admire us…” (Grace, F, 32)

Recovering health and getting back to work and other routines contributed substantially to feelings of ‘normalcy’ in life again. Work was important for material wellbeing and feeling a sense of independence, particularly for men’s fulfilment of their expected masculine role of provider:

**“…**Being at work… it has sustained me because I do not get financial worries. I think that would have disturbed me being there without any source of income, but now that I have a job, it has helped me a lot to live a life today” (Matthew, M, 51).

Work was also very important for psychological wellbeing: it had a restorative role, helping participants to feel that they were restoring what had been lost, and regaining order in their lives, after the earlier losses caused by HIV. It also had therapeutic value, helping people to stay busy and ‘reduce negative thoughts’:

“I am not thinking a lot and not getting worried because of my work of digging, looking after animals. I have goats, pigs and hens. So all my thoughts are on this work… I do not take time to be worried that I am sick…” (Angelo, M, 47).

“She feels proud of her chickens and she takes care of them whole heartedly and that’s why she doesn’t fall sick… when she is talking about it (her business), she is happy… While taking care of the chicks, she told me that even when she finishes feeding them, she sits there. “I love my chicks and during this stage, I cannot sit in my house or sleep for long hours. I make sure I look after them well and this makes me very busy and the day can go unnoticed… Working for myself has helped me so much during this period of “sickness” and I don’t worry about anything. I want to tell you that I am not even aware of the illness” (Linda, F, 29).

#### Adjusting to the new ‘self’: dealing with stigma

A second adjustment process involves coming to terms with a changed self and identity arising from the condition [10]. Processes of reduced self-stigmatisation were evident in all the narratives and an important part of a positive ‘adjustment to a new self’. The reconceptualization of HIV as a normal disease and the normalisation of life discussed above helped participants reappraise their identity as a ‘normal’ person. More importantly, the very visible recovery of their health enhanced self-esteem and reduced self-stigma:

“It’s unfortunate that I don’t have any photos near here, but in those days when I had just tested, I would fear to sit in a congregation or I would feel small whenever I would meet with other people. But nowadays I no longer care because I don’t carry any sign of HIV. I don’t care being looked at. But in the past I used to be suspicious whenever someone looked at me because I had lost weight and used to cough…I would think ‘they are saying I am infected” (Ruth, F, 58).

Health workers offered ways of thinking to resist stigmatisation, encouraging participants to make social comparisons between themselves and *the many others who had not gone for a test and were ignorant of their status*. They viewed themselves, individually and notably as part of a wider group, as knowledgeable and responsible citizens who had taken action to get tested, gain control of the situation, and not harm others:

“They (the health workers) told us that we were better than those who had not bothered to know their status, that we were better than those that were laughing at us…saying that the (TASO) motorcycle has come to your home…they are also sick but do not take the responsibility to get tested so they don’t know their status. That is what made me brave (Judith, F, 27).

This categorisation of themselves as ‘responsible’ citizens compared to ‘others’ who are irresponsible was an in-group identity which enhanced their sense of moral upstanding and self-esteem. Such labelling into ‘good’ and ‘bad’ people has the potential to be divisive and the basis of stigmatisation, but this particular ‘them and us’ distinction was a defence of all HIV patients who were vulnerable to stigma, and a form of resistance thinking against a dominant group in the community who seek to stigmatise.

#### Finding meaning

Several psychological adjustments were evident in the narratives which reflected the three ‘response shift’ mechanisms noted in the introduction. The vast majority enthusiastically spoke of their new appreciation of life, a life which they had thought would soon be over. This was a chance to reappraise, recalibrate the value of health and life, and closely related to this, to see health as a more important priority than other domains in life. Health and life would not be taken for granted:

“Whenever I sleep, I say thank you God for the new day that I am still alive; let me go and see what to do” (Peter, M, 38).

”…I am not saying that I am going to die soon, but I have to plan and also work hard. I actually know that life is short and I have to utilize this chance” (Dorcas, F, 42).

Some participants had more radically reconceptualised what is important for wellbeing, motivated by a new sense of purpose and opportunities for personal growth. Some had decided to support others with HIV, at the clinic or as ‘experts and advisers’ in their community. Most had new plans of hard work and investment in order *to leave something for the children*. Several participants had given more weight and meaning to religion in their life. In particular Evangelical Christianity, popular in this setting, offered a powerful idiom harnessed by some to describe their ‘resurrection’ on ART following a near death experience, and their adjustment or ‘conversion’ to a new and better way of life on ART, which had helped their coping, adjustment and wellbeing.

## Discussion

Our study contributes to knowledge and understanding about PLWH’s self-management work and experiences on ART in a resource-limited setting in Sub-Saharan Africa, and some of the factors which contributed to this group’s self-management successes and wellbeing. Our findings also complement other research showing enhanced wellbeing among PLWH on ART in resource-limited settings [9,14,26–28]. They also offer plausible explanations for recent findings which show higher wellbeing among PLWH on ART compared to others not identified as living with HIV (15,18].

Two cautions about these findings of effective self-management need noting. First, as noted in the first results section, our sample’s overall sense of self-efficacy and successful self-management do not reflect many other PLWH’s experiences in this setting. Second, most of the participants had been on treatment for 2–3 years and were still experiencing a motivation to self-manage and enhanced wellbeing possibly derived from recovery and psychological benefit finding mechanisms. Over time this ‘treatment optimism’ can transition to ‘treatment fatigue’ and reduced satisfaction levels if expectations for life are not met [14,24].

A range of self-management resources, tasks, skills and adjustment processes were identified. The majority of these indicate the importance of social and emotional processes, and a sense of control or self-efficacy in dealing with the condition. Three themes which to differing degrees cut across the three domains of self-management particularly stood out.

First, caring relationships were highly important for self-management and are worth emphasising given that self-management, adherence and wellbeing are often examined with a focus on individual-level determinants [29]. Positive relationships with health workers were particularly important for helping participants to reconceptualise or ‘reframe’ HIV more positively, set them on self-management pathways and resist stigma [30]. These positive relationships enabled a receptiveness to three key resources:

- knowledge about the condition and its treatability, which helped psychological coping and built hope for the future
- practical advice and skills which gave a framework for daily self-management tasks and enhanced a sense of purpose and control
- new ways of thinking and speaking about being HIV positive which helped participants adjust to the condition and their new ‘self’.

Our study also demonstrates the importance of a sense of relatedness to a wider ‘therapeutic community’ of PLWH for self-management and wellbeing [25]. Peer support from other patients at the clinic, and a sense of belonging to a group of PLWH who shared the same experiences and who one could go to for support, were important factors. An in-group identity can bring psychological benefits [31], and more social contact because of HIV has been shown to increase quality of life among PLWH on ART (compared to HIV negative counterparts) in South Africa [16], and in Uganda research shows the positive impact of social support on quality of life among people on ART [32].

Second, participants’ placed great value on new ways of thinking or living life which had arisen from their condition and recovery. They noted spiritual and moral values, and learning about practical self-management, as guidelines for living. Many participants derived a sense of wellbeing from living in accordance with these values and guides. They derived a sense of control from following the guidance of health workers for example, and a sense of achievement from recovering their health. These findings match research which shows that spirituality and religion enhance wellbeing among PLWH [33]. More generally behaving in accordance with values or goals can offer meaning and give greater satisfaction with life despite other adverse circumstances such as low income [24].

A greater appreciation of health and one’s situation also derived from psychological adjustments, such as ‘before and after treatment’ contrasts, which match closely the ‘meaning making’ or ‘benefit finding’ processes identified by Sharpe and Curran [12] and Schulman-Green et al. [10], and processes of post-traumatic personal growth identified elsewhere in the literature [34,35].

Third, and closely related to the above two themes, throughout the findings an ongoing process of adjustment was evident, beginning with adjustment to seeing the condition as treatable and not a death sentence, which gave hope and courage to go on, and moving to recovery of health and ‘looking normal’, which boosted self-esteem, reduced stigma and enabled participants to get on with ‘normal life’. These adjustments were not necessarily always one way and positive, because people faced health-related, economic and emotional set-backs, but in general a process of ‘normalisation’ of the condition and their situation was being described.

The ‘normalisation’ HIV and of life on ART has been identified in other studies in the region [36], but this interpretation requires caution: daily life is medicalised and HIV is a contagious condition which remains stigmatised, and so a particularly difficult chronic condition to manage [2,3]. The struggles of poverty also tempered wellbeing for poorer men and women. As health returned poverty became more a greater source of stress than illness. In fact it was different levels of economic struggle among the participants which most differentiated their narratives about wellbeing, a situation found in other studies [19,26].

The different factors enhancing self-management and wellbeing among this group of people in a resource-limited setting indicate the importance of context and adapting ART self-management frameworks and guidelines to local circumstances to meet people’s needs [8]. This might include: training and supervision to improve inter-personal quality of care and patient-provider relationships; offering simple messages which help people to see HIV as a treatable condition and which offer hope for the future; practical guidance which helps people feel a sense of control over their condition; praise achievements; encourage more peer support at clinics and in the community, and support a sense of ‘therapeutic community’; and perhaps also help people to manage other life challenges. ART programmes need to sustain or strengthen these relational and non-medical aspects of interventions. These aspects of self-management are also likely to be important for other chronic disease interventions in resource-limited settings, at a time when the prevalence of non-communicable diseases such as hypertension is rising.
